# Self-Prioritization Reconsidered: Scrutinizing Three Claims

**DOI:** 10.1177/17456916221131273

**Published:** 2022-11-10

**Authors:** Marius Golubickis, C. Neil Macrae

**Affiliations:** School of Psychology, University of Aberdeen

**Keywords:** self, social cognition, self-prioritization, decision-making

## Abstract

Such is the power of self-relevance, it has been argued that even arbitrary stimuli (e.g., shapes, lines, colors) with no prior personal connection are privileged during information processing following their association with the self (i.e., self-prioritization). This prioritization effect, moreover, is deemed to be stimulus driven (i.e., automatic), grounded in perception, and supported by specialized processing operations. Here, however, we scrutinize these claims and challenge this viewpoint. Although self-relevance unquestionably influences information processing, we contend that, at least at present, there is limited evidence to suggest that the prioritization of arbitrary self-related stimuli is compulsory, penetrates perception, and is underpinned by activity in a dedicated neural network. Rather, self-prioritization appears to be a task-dependent product of ordinary cognitive processes.

The ability to detect self-relevant stimuli is a fundamental social-cognitive ability (e.g., Alexopoulos et al., 2012; Bargh & Pratto, 1986; Gray et al., 2004; Keyes & Brady, 2010; Shapiro et al., 1997; Tacikowski & Nowicka, 2010). This, of course, is no great surprise because failure to identify one’s boss, toothbrush, or cell phone would prompt a host of undesirable outcomes, ranging from outrage and anger to embarrassment and censure. Quite naturally, therefore, through heightened familiarity, personal items are privileged during information processing and response generation, a phenomenon termed “self-prioritization.” Functioning in this way, self-association affords substantial benefits during both thinking and doing (Baumeister, 2022).

Such is the potency of self-relevance, however, it has recently been argued that, following association with the self, even arbitrary stimuli yield comparable advantages. That is, just as one’s mother, bicycle, and laptop are advantaged during stimulus processing, so too are entirely inconsequential items, such as shapes, lines, and Gabor patches. Furthermore, among other things, these self-prioritization effects are claimed to be stimulus driven (i.e., automatic), grounded in perception, and realized in a specialized neuroanatomical network (Humphreys & Sui, 2016; Sui & Humphreys, 2015, 2017). But is this in fact the case? Given long-standing theoretical debate about the nature of self-function (Baumeister, 2022; Dennett, 2003; Gillihan & Farah, 2005; Humphreys & Sui, 2016; Northoff, 2016; Oakley & Halligan, 2017), here we scrutinize these claims and, on the basis of the available evidence, challenge this viewpoint. Although self-relevance undeniably exerts a powerful influence on information processing, we contend that, at least at present, there is limited evidence to suggest that the prioritization of arbitrary stimuli is obligatory, penetrates perception, and is supported by a bespoke neural architecture. Indeed, rather than representing a specialized psychological process, self-prioritization can best be characterized as a task-dependent product of ordinary cognitive operations (Greenwald & Banaji, 1989; Klein & Loftus, 1988).

An inspection of the applicable literature reveals that two paradigms have dominated work exploring the effects of self-association on the processing of arbitrary stimuli—shape-label matching and object-categorization tasks (e.g., Constable et al., 2019; Golubickis et al., 2017, 2018; Schäfer et al., 2016; Sui et al., 2012; Woźniak & Knoblich, 2019). In the popular shape-label matching task devised by Sui and colleagues (Sui et al., 2012), following the association of geometric shapes with the self and various targets of comparison (e.g., self = triangle, friend = circle, stranger = square), participants are required to report whether shape-label stimulus pairs (e.g., square + self, circle + friend, triangle + stranger) correspond with the previously learned associations (Schäfer et al., 2016; Sui et al., 2012; Woźniak & Knoblich, 2019). Relatedly, in object-categorization tasks, after the assignment of arbitrary stimuli to the self and a familiar other (e.g., self owns pens, friend owns pencils), participants must indicate to whom exemplars from each class of item belong (Golubickis et al., 2018, 2021). Importantly, these tasks generate robust self-prioritization effects (i.e., faster responses to stimuli that represent, or are possessed by, the self compared with others) and serve as the foundation on which claims about the characteristics and origin of self-bias have been based (Humphreys & Sui, 2016; Sui & Humphreys, 2015, 2017).

Given their operational characteristics, however, shape-label matching and object-categorization tasks are poorly suited for establishing the automaticity and perceptual basis of self-prioritization. Indeed, on the basis of the available evidence, rather than reflecting an obligatory stimulus-driven product of early perceptual processes, here we argue that self-prioritization derives from task-dependent differences in the efficiency of the memorial and attentional processes that support decision-making (D’Esposito & Postle, 2015; Oberauer, 2019). Operating in tandem, working memory and attention serve as the mechanisms through which stimuli are selected and prioritized for subsequent processing. In this regard, the ease with which powerful self-item (vs. friend-item or stranger-item) associations can be formed naturally facilitates both the maintenance of these representations in working memory and the allocation of attention to related material during decisional processing (Oberauer, 2019). As effective search templates in working memory, self-object (vs. other-object) associations guide attention (i.e., preferential allocation of cognitive resources) to matching stimuli, thereby facilitating task performance (Awh et al., 1998; Janczyk et al., 2019; Olivers et al., 2011; Reuther & Chakravarthi, 2017; Soto et al., 2008; Yin et al., 2019). Recently, in a delayed match-to-sample spatial working-memory task, Yin et al. (2019) demonstrated just such an effect. Participants responded faster to working-memory probes when they appeared at locations containing a self-associated color (see also Yin et al., 2021) compared with arbitrary colors paired with other persons (i.e., friend and stranger).

The key status of self-shape (or self-object) associations in working memory is highlighted further by the difficulties that are encountered when these relations are no longer applicable (i.e., task-relevant). Using a relearning paradigm, Wang et al. (2016) showed that when previous shape-label associations were switched such that a stimulus formerly paired with the self or a friend had to be associated with a stranger, performance costs were observed. Specifically, shape-label matching impairments were most pronounced when a new association involved the stimulus previously linked with the self compared with a friend. Thus, although self-object associations in working memory routinely facilitate processing, under certain conditions these powerful (i.e., sticky) relations can also disrupt decision-making (Golubickis & Macrae, 2022; Yin et al., 2019).

Through the repeated experience of forming self-object linkages in everyday life, it is unsurprising that arbitrary stimuli routinely benefit from self-association during shape-label matching and object-categorization tasks. Following connection with the self, personal relevance exerts a powerful although ordinary influence on the memorial and attentional operations that underpin decisional processing (Greenwald & Banaji, 1989; Klein & Loftus, 1988). Based on these observations, our objective in the current article is to highlight that, despite dominating contemporary theorizing on the topic (Humphreys & Sui, 2016; Sui & Humphreys, 2015, 2017) there is at present limited evidence to suggest that the prioritization of arbitrary self-related material is obligatory, grounded in perception, and supported by specialized processing operations.

## Is Self-Prioritization Obligatory?

A defining characteristic of an automatic psychological process is that it is stimulus driven, requiring neither intention nor instruction to occur and, once set in motion, unstoppable (Bargh, 1989; Moors & De Houwer, 2006). According to prominent accounts of self-function, self-prioritization arises in just such a way, such that the mere registration of personally relevant material is sufficient to trigger stimulus enhancement. As Sui et al. (2014) put it, “self-biases can emerge . . . from automatic bottom-up biases” (p. 1183). Although this may well be the case, this conclusion cannot be drawn from the self-prioritization effects that have been demonstrated using standard shape-label matching and object-categorization tasks (Caughey et al., 2021; Constable et al., 2019; Dalmaso et al., 2019; Falbén et al., 2019; Siebold et al., 2015; Stein et al., 2016; Wade & Vickery, 2017; Woźniak & Knoblich, 2019). Muddying the interpretational waters are the processing objectives essential to the successful completion of these activities. To respond accurately in each of these paradigms, participants must explicitly consider the self-relevance (or otherwise) of the to-be-judged stimuli (i.e., whom the shape denotes, who owns the object). Given this requirement, it is not possible to conclude that speeded responses to self-relevant (vs. other-relevant) items reflect the automaticity of self-prioritization. Put simply, one cannot infer that self-prioritization is driven by bottom-up stimulus-based processes when the task at hand is to judge the personal relevance of the presented shapes or objects (Golubickis et al., 2018; Sui et al., 2012).

Further undermining the putative stimulus-driven basis of self-prioritization is the demonstration that processing benefits fail to emerge when personally associated items are appraised along dimensions unrelated to self-relevance (e.g., Caughey et al., 2021; Constable et al., 2019; Dalmaso et al., 2019; Falbén et al., 2019; Siebold et al., 2015; Stein et al., 2016; Wade & Vickery, 2017). For example, using a shape-classification task, Caughey et al. (2021) demonstrated that self-relevance facilitated performance only when task instructions directed attention to previously formed target-shape associations in working memory (i.e., reporting to whom the shape, i.e., self or friend, referred). When, instead, emphasis switched to reporting the identity of the shape (i.e., triangle or square) or its spatial location (i.e., above or below fixation), self-prioritization failed to emerge. Likewise, in the context of personal possession, when required to report which of two objects (i.e., mugs) initially appeared on the computer screen (i.e., temporal-order judgment task), Constable et al. (2019) observed that participants were biased toward reporting that self-owned (vs. experimenter-owned) items were presented first. This effect was abolished, however, when the requested judgment probed whether the object seen first appeared to the left or right of fixation. Findings such as these are inconsistent with the contention that the mere registration of personally relevant items is sufficient to elicit self-prioritization (Sui et al., 2014).

Corroborating and extending work of this kind, a recent investigation cleverly manipulated learning instructions to make participants believe that self-association was either a relevant or irrelevant aspect of the shape-label matching task they were performing (Woźniak & Knoblich, 2022). Critically, stimulus prioritization emerged only when self-association was assumed to be task-relevant. In other words, self-prioritization was driven not by the strength of self-association but rather how the task was conceptualized by participants. Collectively, these studies provide valuable insights into the conditions under which self-relevance facilitates the processing of arbitrary stimuli (Caughey et al., 2021; Constable et al., 2019; Falbén et al., 2019; Woźniak & Knoblich, 2022). In contesting the stimulus-driven basis of self-bias (Humphreys & Sui, 2016; Sui & Humphreys, 2015, 2017), it is apparent that prioritization is moderated by the extent to which cognitive resources are directed to target-item associations in working memory during decisional processing. Only when these associations are made salient (or deemed task-relevant) does self-prioritization arise.

The inherent malleability of stimulus prioritization is further evidenced by the demonstration that comparable effects can be generated for targets other than the self (i.e., stimulus prioritization is not exclusive to the self). A feature of standard shape-label matching and object-categorization tasks is that no other task-related information is provided to participants (Golubickis et al., 2018; Sui et al., 2012). For example, the composition of the to-be-judged sample of shapes or objects is unspecified. This contrasts with processing outside the laboratory in which powerful expectations signal the likelihood of encountering self-related stimuli in various settings (e.g., one’s own apartment compared with a friend’s apartment). Under conditions of stimulus uncertainty, it is probable that item relevance serves as the most prominent aspect of the immediate task context (e.g., I have been associated with a shape or object), thereby triggering self-prioritization (Golubickis et al., 2018; Golubickis & Macrae, 2021; Sui et al., 2012). In other words, self-bias emerges as a strategic response to the prevailing task conditions rather than as an obligatory stimulus-driven product of social-cognitive functioning.

Supporting this viewpoint, recent research has demonstrated that people prioritize whichever material is most salient/goal-relevant in the current task context, potentially at the expense of self-related stimuli (Cunningham et al., 2022; Falbén et al., 2020; Svensson et al., 2022; Woźniak & Knoblich, 2022). For example, whether in an expectancy-confirmatory or expectancy-violating task setting, prioritization effects emerge for whichever stimuli (i.e., self-related or friend-related) are encountered most frequently in both shape-label matching and object-categorization tasks (Falbén et al., 2020; Svensson et al., 2022). Thus, at least where arbitrary stimuli are concerned, processing is facilitated for goal-relevant material regardless of the target with whom the items have been associated. Again, this speaks against the stimulus-driven basis of self-prioritization. In terms of magnitude, however, it should be noted that prioritization effects are typically larger for material associated with the self than comparable items paired with others (e.g., best friend), a finding that derives from differences in the efficiency of the component processes (i.e., access to working memory, template matching) that underpin task performance during decision-making (Svensson et al., 2022; Yin et al., 2019, 2021).

Moving beyond shape-label matching and object-categorization tasks, several studies have explored the automatic prioritization of arbitrary stimuli in paradigms better suited to probe this topic (e.g., cueing tasks). Given its pivotal status in social-cognitive functioning, it has been argued that self-relevance influences three core facets of attention (Sui & Rotshtein, 2019)—alerting, orienting, and executive control (Corbetta & Shulman, 2002; Petersen & Posner, 2012; Posner et al., 2016; Posner & Petersen, 1990). Work substantiating this claim, however, is limited. Whereas Sui et al. (2009) failed to observe a reflexive orienting effect when arrows were associated with self (vs. friend) and Orellana-Corrales et al. (2020, 2021) found no effect of newly self-associated (vs. other-associated) stimuli on attentional capture using a dot-probe task, Macrae et al. (2018) demonstrated that, through their effects on transient attention, self-relevant (vs. other-relevant) cues enhanced contrast sensitivity. Likewise, although Li et al. (2022) reported the benefits of self-relevance on alerting and orienting using a revised version of the Attention Network Task, Svensson et al. (2022) found that only executive control was sensitive to the personal significance of stimuli, such that conflict resolution was enhanced after the presentation of self-associated compared with friend-associated cues (see also Golubickis & Macrae, 2021). Collectively, these findings yield inconclusive evidence that self-relevance automatically facilitates all stages of attentional processing.

Tempting though it may be to conclude that self-relevance benefits the processing of arbitrary items in an automatic fashion (Humphreys & Sui, 2016; Sui & Humphreys, 2015, 2017), evidence supporting this viewpoint is scarce. Rather than comprising an obligatory social-cognitive operation, stimulus prioritization is manifestly sensitive to people’s goals, expectations, beliefs, and the probability that self-relevant (vs. other-relevant) material will be encountered, a reflection of its task-dependent psychological status (Caughey et al., 2021; Constable et al., 2019; Cunningham et al., 2022; Golubickis & Macrae, 2021; Svensson et al., 2022; Woźniak & Knoblich, 2022). Of course, in no way does this rule out the possibility that self-relevance could conceivably exert an obligatory stimulus-driven influence on the processing of arbitrary material; it is simply that, at present, compelling evidence for the operation of such an effect is conspicuously absent. Additional research is required to clarify this matter.

## Does Self-Prioritization Penetrate Perception?

According to dominant theoretical accounts, self-prioritization wields considerable influence on perceptual processing (Humphreys & Sui, 2016; Sui & Humphreys, 2015, 2017). Endorsing the viewpoint that perception is cognitively penetrable (Clark, 2003; Lupyan, 2015; Vetter & Newen, 2014) assumes that, much like other top-down influences (e.g., beliefs, desires, preferences), self-relevance causally influences the contents and character of people’s visual experiences (Bruner, 1957). As Humphreys and Sui (2016) stated, “Relating a stimulus to the self (self-reference) enhances perception” (p. 719). This general topic, of course, has attracted significant debate over the years, with many challenging the cognitive-penetrability hypothesis (Firestone & Scholl, 2016; Fodor, 1983; Pylyshyn, 1999; Raftopoulos, 2009). Central to this competing account is the belief that sensory inputs are translated into fixed neural codes in which the corresponding stimulus representations have not been modified in any way by higher-order cognitive factors. In other words, stimuli are perceived independently of people’s motivations, expectations, and emotional states (i.e., perception is encapsulated).

Based on this line of thinking, rather than representing an objective assessment of a percept (e.g., the color of an apple), myriad cognitive-penetrability effects turn out to be confounded by subjective interpretational/inferential influences (e.g., the price of an apple) that are linked to the operation of postperceptual attentional, memorial, and judgmental processes (Firestone & Scholl, 2016). Not without controversy (Clark, 2003; De Lange et al., 2018; Lupyan, 2015; Lupyan et al., 2020; Vetter & Newen, 2014), this viewpoint is nevertheless useful because it provides criteria for establishing what constitutes convincing evidence for the influence of top-down effects on early visual processing. In this regard, and with specific reference to social-cognitive functioning, Firestone and Scholl (2016) identified several questions that must be considered before concluding that cognition impacts perception. Notably, do the observed effects reside in differences in perceptual or judgmental processes? Is it possible the findings are driven by demand effects or response biases? Are perceptual and memorial processes conflated in the experimental tasks of interest? Crucially, on inspection of the available evidence, these issues have direct implications for the supposed effects of self-association on perceptual processing.

To date, research proposing that self-relevance penetrates perception comes largely from a small number of studies demonstrating that self-associated (vs. other-associated) stimuli yield faster and more accurate responses in variants of the shape-label matching task (Sui, Enock, et al., 2015; Sui et al., 2012, 2013; Siu, Liu, et al., 2015). For example, if self-relevance enhances early vision, Sui et al. (2012) reasoned that reduced stimulus detectability (i.e., high vs. low luminance contrast) should exert a less detrimental impact on the processing of self-associated compared with friend-associated stimuli during shape-label matching. Observing this effect, they took this as confirmation that self-relevance infiltrates perception. Two issues merit consideration, however. First, in more recent research, this effect has failed to replicate (Scheller & Sui, 2022). Second, and more generally, tasks that do not probe the appearance of a stimulus but rather tap conceptual aspects of the item confound perceptual and memorial processing (Sui et al., 2012, 2013). To categorize a stimulus, one must first establish whether current visual inputs resemble any stored mnemonic representation (i.e., categorization entails stimulus recognition). With precisely this prerequisite, shape-label matching tasks (or indeed any categorization/classification task) cannot therefore be used to assert, unambiguously, that self-association penetrates early visual processing (Firestone & Scholl, 2016).

Of the handful of experiments that have explored self-prioritization in tasks that do not require stimulus recognition (Caughey et al., 2021; Constable et al., 2019; Dalmaso et al., 2019; Falbén et al., 2019; Siebold et al., 2015; Stein et al., 2016; Wade & Vickery, 2017), none have adopted measures that exclusively tap perception. Instead, tasks have been used in which a combination of perceptual and attentional enhancements during stimulus processing influence performance (Macrae et al., 2018). Significantly, this work has yielded limited evidence that self-association impacts what people see. For example, using breaking continuous flash suppression to examine the ease with which items (i.e., Gabors) associated with the self and a target of comparison access visual awareness during a stimulus-localization task (i.e., in which of four quadrants did a stimulus appear), Stein et al. (2016) observed no effect of self-association on the time taken for stimuli to overcome interocular suppression. In a similar task context, in contrast, when stimulus recognition (i.e., self or friend) was the central component of the to-be-completed activity, the benefits of self-association were observed (i.e., self-associated Gabors entered consciousness most rapidly; Macrae et al., 2017), an effect that was driven by a decisional preference for self-related responses before stimulus presentation (Firestone & Scholl, 2016).

As noted earlier, rather than originating in perception, the self-prioritization effects observed in standard shape-label matching and object-categorization tasks are underpinned by differences in the efficiency of the cognitive operations that support decision-making (D’Esposito & Postle, 2015; Oberauer, 2019). Interestingly, however, the observation that self-object associations in working memory serve as effective search templates speaks to the possibility that top-down influences could potentially penetrate early perceptual processing (Clark, 2013; Lupyan, 2015; Vetter & Newen, 2014). Disambiguating sensory regions from the neural pathways underpinning working memory is challenging because in many tasks these areas overlap considerably (Cappotto et al., 2022; Christophel et al., 2017). Take, for example, recent research by Yin and colleagues (2021). Using functional MRI to explore the neural mechanisms that support the prioritization of arbitrary self-related material in spatial working memory, Yin et al. (2021) reported several interesting effects. First, maintaining self-related (vs. other-related) items increased activity in prefrontal cortex and components of the working-memory network, notably the superior parietal lobule. Second, using multivoxel pattern analysis, this elevated activity when maintaining self-related (vs. other-related) items was accompanied by the enhanced representation of spatial locations corresponding to self-associated stimuli in the visual cortex. At least in the context of spatial working memory, this then may serve as a pathway through which self-relevance influences visual processing. During matching tasks, potent self-associated search templates send projections to primary/secondary visual regions, thereby enhancing perception (Kok et al., 2014).

Notwithstanding repeated claims about the origins of self-prioritization, at present there is a dearth of evidence to suggest that self-relevance penetrates perceptual processing. The primary difficulty is that, in the extant literature, studies have explored stimulus recognition rather than people’s reports of what they have seen (Golubickis et al., 2018; Sui et al., 2012, 2013). To overcome this limitation, care must therefore be taken to distinguish between perception and memory through the adoption of dependent measures that isolate the specific perceptual effects of interest, a challenge that is also present in related social-cognitive work (e.g., emotion and perception; see Niedenthal & Wood, 2019). This of course is no easy undertaking because what constitutes a pure measure of perception remains open to debate. At the very least, however, tasks should be adopted that use appropriate stimulus materials and measure the contents of perception rather than the products of computations that have been undertaken on sensory inputs (Firestone & Scholl, 2016). In this regard, there are several potential candidates.

Using arbitrary stimuli (e.g., shapes, objects, Gabors) that have been systematically altered along basic physical dimensions (e.g., color, brightness, contrast, frequency, signal-to-noise ratio; Gescheider, 2013), we suggest three methodologies that can be adopted to explore the effects of self-association on perception: first, magnitude estimation in which participants rate the visual characteristics of stimuli (e.g., brightness of self-related vs. other-related items; Narens, 1996); second, tasks in which participants adjust one of two stimuli until they look identical on a visual dimension of interest (e.g., color, contrast; Gilchrist et al., 1999); and third, detection and discrimination tasks in which participants detect small differences between stimuli using methods of adjustment or present/absent judgments (Ratcliff, 2002). Methods such as these are useful because the thresholds of sensory detection can be estimated separately for various person-item associations (e.g., self-relevant vs. friend-relevant), thus establishing the extent to which personal significance facilitates low-level perceptual processing (Humphreys & Sui, 2016; Sui & Humphreys, 2015, 2017).

## Is Self-Prioritization Supported by a Specialized Processing Network?

Prominent in recent theoretical writings is the contention that distinct patterns of neural activity underpin the prioritization of arbitrary self-associated stimuli. For example, according to Humphreys and Sui (2016), a dedicated self-attention network (SAN) supports responses to personally related (vs. other-related) stimuli (including arbitrary items) through the interaction of three cortical nodes: a self-relevance hub located in the ventromedial prefrontal cortex (vMPFC), a top-down attentional-control circuit involving the dorsolateral prefrontal cortex and intraparietal sulcus, and a bottom-up attentional-orienting mechanism situated in the posterior superior temporal sulcus (pSTS). In essence, the vMPFC signals the presence of self-relevant stimuli, with the consequent interplay between top-down and bottom-up attentional operations enhancing the salience of this material. Thus, underpinned by specialized neurocognitive operations, self-prioritization is psychologically special.

Interactions between areas of the SAN—most notably the vMPFC and pSTS—have served as the bedrock for claims about the nature and origin of self-prioritization. For instance, Humphreys and Sui (2016) proposed that “nodes that respond to self-related stimuli (the vMPFC and pSTS) interact with nodes within an attentional control network to determine perception and action” (p. 15). It is somewhat puzzling, therefore, that early visual areas do not comprise components of the SAN, and, as noted previously, tasks that tap the effects of self-association on perceptual processing have not yet been adopted to test the model (Firestone & Scholl, 2016). In addition, in a bold statement regarding the character of social-cognitive functioning, Humphreys and Sui (2016) further suggested that “interactions between these processing nodes determine our response to stimuli linked to the self rather than other people” (p. 11). In other words, the network is self-specific. Although we do not dispute that, at least in certain task contexts, the SAN is preferentially involved in the processing of self-relevant (vs. other-relevant) material, echoing our other observations, we contend there is insufficient evidence to conclude that this network is dedicated to self-referential processing.

Despite numerous attempts to chart how self-association exerts a unique influence on stimulus prioritization, evidence suggesting the operation of a dedicated processing network remains elusive (Mainz et al., 2020). For example, although Sui et al. (2013) observed increased activity in both the vMPFC and pSTS during a shape-label matching task, only self-related and stranger-related responses were discriminable in the prefrontal cortex. Crucially, responses to self-related and friend-related stimuli were equivalent, thus suggesting this region was sensitive to material pertaining to any familiar target (Gillihan & Farah, 2005). Likewise, in patient research, Sui, Enock, et al. (2015) reported that whereas damage to the vMPFC and insula attenuated self-bias (vs. stranger) relative to control participants, impairment to the temporal lobe increased the prioritization of self-relevant material. Critically, however, again no differences between self and a familiar target (i.e., friend) were observed. In addition, adopting a global/local stimulus-identification task to explore the neural correlates of self-bias, Sui, Liu, et al. (2015) reported no differences in activity in either frontal or temporal regions as a function of target-stimulus association. Finally, investigating the relationship between self-referential and emotional processing, Yankouskaya and Sui (2021) observed that activity in the vMPFC was similar for both self-associated and positive stimuli.

Further weakening the alleged causal connection between the vMPFC and self-bias, recent work has demonstrated that disrupting this region through transcranial direct-current stimulation does not always abolish self-prioritization. For example, using a shape-label matching task, both Schäfer and Frings (2019) and Martínez-Pérez and colleagues (2020) reported that stimulating the vMPFC did not eliminate the emergence of the standard self-prioritization effect. It should be noted, however, that during a spatial variant of the matching task, stimulating the vMPFC has been reported to eradicate the prioritization of arbitrary self-relevant stimuli in working memory (Yin et al., 2021). Thus, consistent with the SAN model (Humphreys & Sui, 2016), this provides initial evidence that, at least during a spatial working-memory task, the vMPFC is causally implicated in the emergence of self-bias.

As others have noted, however, it is questionable whether self-bias can be attributed to the operation of specialized operations in a dedicated neuroanatomical network (Gillihan & Farah, 2005; Greenwald & Banaji, 1989). In particular, the claim that self-specific information is supported by a dedicated hub, housed in the vMPFC, lacks functional specificity (García et al., 2015). Extensive research has shown that this cortical region is engaged during a wide range of tasks, including (but not limited to) decision-making, emotion regulation, memory retrieval, and learning (Hiser & Koenigs, 2018). Given the component processes involved in shape-label matching and object-categorization tasks, the vMPFC may therefore be responsive to a range of factors other than self-association. For example, activity may reflect the maintenance of goal states, the anticipation of specific outcomes, or the manipulation of decision-relevant material (Euston et al., 2012; Walton et al., 2015). Thus, although supporting core components of social cognition (Amodio & Frith, 2006; Lieberman et al., 2019), aside from recent research by Yin and colleagues (2021), there is limited evidence to suggest that the vMPFC plays a pivotal role in the generation of self-prioritization effects, at least for arbitrary stimuli.

Rather than relying on specialized operations, self-prioritization likely resides in the workings of a domain-general neuroanatomical network. Previous research has highlighted the engagement of regions of the frontoparietal cortex across a variety of tasks (Corbetta & Shulman, 2002). Specifically, top-down signals from the prefrontal and parietal cortices strengthen the processing of task-relevant stimuli (Buschman & Kastner, 2015), notably the selection and maintenance of information in working memory (Myers et al., 2017). It is well established that, based on material held in temporary storage, this frontoparietal (i.e., executive) network underpins goal-directed processing, including rule-based problem-solving and goal-oriented decision-making (Dixon et al., 2018; Menon, 2011; Uddin et al., 2019). With precisely these characteristics, the decision-making tasks that reliably yield self-prioritization effects (or for that matter friend-prioritization effects; Falbén et al., 2020; Svensson et al., 2022) should be supported by activity in this frontoparietal attentional system. Operating in this way, self-prioritization would be analogous to the enhancement of any class of stimuli for which active and effective search templates in working memory direct attention (i.e., cognitive resources) to goal-relevant material during decisional processing (Olivers et al., 2011; Soto et al., 2008; Wade & Vickery, 2017).

To elucidate the neural correlates of self-function, a useful task for future research will be to contrast self-bias with the prioritization effects that have been reported for other targets (Falbén et al., 2020; Svensson et al., 2022). As previously noted, when no other information is provided, participants default to the most salient aspect of the task context (e.g., I am a triangle, I own pens; Golubickis et al., 2018; Sui et al., 2012), thereby triggering self-prioritization. When, however, new search templates are induced through the provision of additional task-related details (e.g., friend-associated items predominate during the task; Falbén et al., 2020; Svensson et al., 2022), self-bias is replaced by friend prioritization. Of theoretical interest, therefore, is whether components of the SAN yield equivalent or distinct patterns of activation during the prioritized processing of arbitrary self-associated and friend-associated material. If, for example, comparable neural effects were observed, this would support the proposition that prioritization is underpinned by a domain-general network in which projections in working guide attention toward goal-relevant stimuli (Olivers et al., 2011; Soto et al., 2008; Wade & Vickery, 2017). In contrast, if these effects diverge in significant ways, this would affirm the unique status of self-function (Humphreys & Sui, 2016).

Another interesting avenue of investigation concerns how self-prioritization compares with the biasing effects that have been observed for other classes of stimuli, such as rewarding, arousing, or emotionally significant material (Carlson & Mujica-Parodi, 2015; Öhman, 2007; West et al., 2009). For example, is self-bias simply a reflection of the reward value or affective salience of arbitrary stimuli that have temporarily acquired personal significance (Blaney, 1986; Northoff & Hayes, 2011; Sui et al., 2016; Yankouskaya et al., 2022)? According to an influential account, processing self-related and reward-related material is inexorably intertwined, thereby suggesting considerable overlap in the neuroanatomical structures that support stimulus prioritization (de Greck et al., 2008; Yankouskaya et al., 2022). Of interest, therefore, would be imaging investigations that explore the extent to which prioritization emerges for whichever stimuli elicit enhanced responses (e.g., expected self-related or friend-related stimuli) and how this relates to reward-related processing in the brain (Clithero & Rangel, 2014; Yankouskaya et al., 2017). Parallel lines of inquiry should also explore the emotional impact of self-associated material. Given the proclivity to construe self-related stimuli positively (Beggan, 1992; Sedikides & Alicke, 2012), it is possible that self-bias is a simple manifestation of affective prioritization, as reflected in the associated patterns of neural activity (Stolte et al., 2017; Yankouskaya et al., 2022).

## Conclusion

With a mind that is exquisitely receptive to personally meaningful material, researchers have probed whether the benefits of self-association extend to arbitrary stimuli (Constable et al., 2019; Golubickis et al., 2018; Sui et al., 2012; Woźniak & Knoblich, 2019). Observing that indeed they do, it has been claimed that, like other manifestations of self-prioritization, this is an obligatory stimulus-driven outcome, grounded in perception, and underpinned by a dedicated processing network (Humphreys & Sui, 2016; Sui & Humphreys, 2015, 2017). Here, on the basis of the available evidence, we scrutinized these assertions and challenged this viewpoint. First, we suggested that self-prioritization, rather than emerging automatically, is inherently task/goal-dependent. Second, we argued that self-relevance, although impacting basic facets of memory and attention, does not penetrate perception. Third, we proposed that self-prioritization does not rely on a bespoke neurocognitive architecture but rather is underpinned by domain-general processes in the brain’s frontoparietal attentional network.

To be clear, we are not suggesting that stimuli with long-standing personal significance (e.g., one’s partner or car or dog) do not exert pervasive effects on multiple stages of information processing. After all, it would be puzzling and counterproductive if the mind operated in this way. The benefits of prioritizing familiar stimuli are considerable (Baumeister, 2022). What we are proposing, however, is that, to date, evidence suggesting that arbitrary self-related material elicit comparable effects is, at best, limited. Given these observations, we eagerly await future lines of investigation to advance understanding of this core social-cognitive topic.

## Transparency

*Action Editor:* Klaus Fiedler

*Editor:* Klaus Fiedler
